# Study of Consumers’ Purchase Intentions on Community E-commerce Platform with the SOR Model: A Case Study of China’s “Xiaohongshu” App

**DOI:** 10.3390/bs13020103

**Published:** 2023-01-26

**Authors:** Baodeng Lin, Binqiang Shen

**Affiliations:** 1 School of Economics and Trade, Fujian Jiangxia Uiversity, Fuzhou 350108, China; 2 Institute of Education, Xiamen University, Xiamen 361005, China

**Keywords:** community e-commerce, purchasing intention, SOR model, perceived value, content marketing

## Abstract

As e-commerce flourishes, a new model known as community e-commerce has sprung up, which gathers users of the same interests and values and provides precise content marketing for specific groups, to satisfy the various demands of customers. Studying the influencing factors of community e-commerce platforms on the purchasing willingness of customers can offer valuable references for a healthily developed community e-commerce. On the basis of the SOR model, this work establishes a structural model of the consumers’ purchase intentions on community e-commerce platforms. Citing the Xiaohongshu application, an analysis is conducted, using structural-equation modeling and questionnaire. The results show that the perceived value is positively influenced by the product features, content marketing, and community factors which prod customers to be ready to purchase. Based on the research conclusions, management suggestions are proposed, including building products with utmost cost–performance, emphasizing precise content-marketing, creating an active atmosphere in the community, and establishing the social responsibility of platforms, etc.

## 1. Introduction

According to the latest data from The 49th Statistical Report on Internet Development in China released by the China Internet Network Information Center (CNNIC), the number of online shopping users in China reached 842 million as of December 2021, 59.68 million higher than that in the same period of last year [1]. It is implied that China has a huge amount of online-shopping users with increasingly richer customer demands. At this time, the development mode of traditional e-commerce has reached its maximum, with waning users, failing to meet the varying purchasing needs of customers. It is during this time that a new model of e-commerce based on user sharing keeps sprouting, that is, cpeople have gradually favored the way of “community+e-commerce”. People actively exchange their user experience of products and services on community e-commerce platforms. Their requirements are satisfied during the communication, thus promoting the development of the community economy. Community e-commerce provides a platform for communication with consumers by linking people with the same interests and preferences, and gathers consumers with consumption potential. Inspired by this, many businesses have started to activate the engagement of lurking users by organizing communities.

With the continuously evolving community economy, more community factors are integrated into the Internet, followed by the sprouting community e-commerce models. Community e-commerce allows more diversified demands to be met and has gained attention from more consumers. The exploration of the factors of community e-commerce platforms that influence consumers’ purchasing intention can provide directions for these platforms and prod them to focus on the products and services characteristics, as well as methods to enrich the content marketing. In this way, the significance of the community can be better understood. By building connections with customers, more accurate services will be provided for users, thus keeping more users coming back and continuously improving sales to expand diversified service demands. By studying the relevant literature, we found that most previous studies on consumers’ purchase intentions are based on the traditional e-commerce platform model and its development. While the new community model has been a hot topic, limited research has comprehensively analyzed its influence on consumers’ purchase intentions. This research takes a typical social e-commerce platform, Xiaohongshu App, as a case study. The purpose is to construct a SOR model to study the characteristics of the marketing model of this platform, based on which an empirical analysis of the factors influencing the purchase intention of consumers on social e-commerce platform was conducted. This study can effectively fill the gaps in the existing literature, and is therefore important. The Xiaohongshu App, the most representative platform of community e-commerce in China, is selected as the study object, and its marketing model is analyzed. Xiaohongshu promotes explosively the community e-commerce model to the public to the maximum extent through its content marketing methods, such as providing tourism strategies and courses for makeup and dressing. The latest data reported by iMedia research shows the number of Xiaohongshu users reached 300 million by July 2019, with a monthly active user rate of more than 100 million, which still shows a growing trend [2]. Therefore, through research and example analyses, management suggestions are given for developing community e-commerce platforms, which will provide important decision-making references for the operation managers of other community e-commerce platforms.

## 2. Literature Review

### 2.1. An Overview of Community E-commerce

#### 2.1.1. Basic Concept of Community E-commerce

Community e-commerce is defined as the e-commerce activities supported by community relations, in which the community needs not only independent self-expression, but also a stable structure and consistent group concepts. As the Internet develops into various fields, the concept of “community” has gradually integrated into business operations, ultimately evolving into a new model of “community+e-commerce”, bringing connections, communications, information, transactions, and other value to sustainable business marketing. Li et al., pointed out that community e-commerce is essentially transaction activities driven by social relationships based on users’ connections [3]. Luo and Li explored the innovation of business models, pointing out that the community-oriented platform model would become a place where e-commerce enterprises and consumers are connected to communicate and create value, which could meet consumers’ deeper level of demands and become a new model for e-commerce development [4]. The most fundamental aspects of the marketing operation of community e-commerce lie in accurate community positioning, in-depth efforts in the pain points of the community, high-quality content and services, emphasis on public praise and influence, improved purchase experience, and the realization of community value. Community e-commerce that emerges in the Internet era can keep abreast of the development trend of the time and is expanded based on traditional e-commerce, which can fully reflect the community economy. Through the community-based transformation of each user, the dynamic mapping ability will be utilized by commercial marketing.

#### 2.1.2. A Summary of Research on Community E-commerce

In an era of a thriving Internet, the traditional e-commerce model has failed to meet the needs of consumers. The diversified demand has driven consumers to form a community group with the same perception, which constantly boosts the development of the community economy, and advances the expansion and reform of e-commerce enterprises. Wang et al., took the Pinduoduo app as an example to analyze its rapid rise and the current dilemma of user traffic facing traditional e-commerce. Their study concluded that traditional e-commerce was a necessary step for the community e-community before its development [5]. Xu and Liu analyzed the differences between community e-commerce and traditional e-commerce in terms of business model, user-traffic characteristics, access to attract users, etc., based on their differences. They pointed out that traditional and community e-commerce should be combined in collaborative development by jointly satisfying consumers’ demand, complementing the ways to attract customers, diversifying the enjoyment of shopping, and improving social attributes, etc., [6]. Wu et al., verified whether precision marketing was effective, using the data from the community e-commerce platforms, and found that sustainably moderate marketing was feasible for community e-commerce [7]. Yin carried out detailed analyses of the advantages and disadvantages of the business model of the community e-commerce platform—Xiaohongshu. They noted that in the context of the current development of community e-commerce, analysis and decisions should be made by community-based thinking, connection, scenes, and content, thus creating an atmosphere where consumers are willing to share and purchase [8]. As a new business model, community e-commerce can integrate the traffic information of consumers and meet their diversified needs, which is not only a supplement to the current traditional e-commerce development but also a new way for e-commerce to progress.

### 2.2. Overview of Consumers’ Purchase Intention

#### 2.2.1. Definition of Consumers’ Purchase Intention

Purchase intention is a consumer’s subjective tendency to choose a product. It is composed of consumers’ attitudes towards specific products and brands, as well as external factors. As a type of consumer psychological activity, it refers to consumers’ willingness and the possibility to purchase a certain product [9]. Research by Dodds et al., indicated that purchase intention presented the subjective tendency of consumers to purchase specified products or services, and could be considered a purchase plan, which was an important indicator for evaluating their subjective needs or affection for goods [10]. The consumer’s willingness is an emotional decision before purchasing, through which customers’ upcoming behavior and purchasing decisions can be inferred. Therefore, a comprehensive understanding of the factors affecting consumers’ purchase intentions will help stimulate their purchase behavior.

#### 2.2.2. Research on Influencing Factors of Consumers’ Purchase Intentions and Social E-commerce Consumers’ Purchase Intentions

The purchase intention depends on external environmental factors and the consumers’ conditions. Evaluating these two types of factors to make decisions is a key area for scholars worldwide. For example, Lai and Peng’s research studied factors affecting the purchase intention of agricultural products based on the double-factor analysis, proving that the consumers’ purchase intention could be stimulated by improving the cost performance of products, the service quality of enterprises, and the shopping environment of platforms [11]. Based on the service-dominant logic and the information-adoption model, Onofrei et al., discussed the relationship between social media and consumers and put forward the idea that positive interaction on social media would elevate consumers’ purchase intentions [12]. Liu and Xu introduced the stimulation-response theory to explore the impact of price judgment on purchase intention, indicating that consumers’ price judgments on products can positively influence the quality of perception, thus prompting consumers’ purchase intentions [13]. Based on consumers, Kim et al., confirmed that the purchase intention was positively correlated with the persistence of consumer perception and inversely correlated with perceived suspicion, by researching the impact of the sustainable perception level of sports-product consumers [14]. The studies mentioned above by related scholars start from both internal and external factors, such as product quality and price, social interaction, consumer-perception persistence, etc., to study the influencing factors of consumer purchase-intention. By combing through the existing literature, this paper classifies the factors influencing the purchase intention of social e-commerce consumers into three categories, namely, product characteristics, content marketing and community factors, which are very specifically outlined in the research hypotheses in the third part of this paper.

### 2.3. Research on Relevant Theories and Models

#### 2.3.1. Stimulation-Organism-Response Model

**Figure 1:**
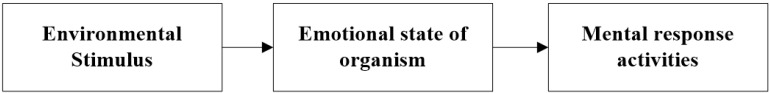
S-O-R model.

In the early 20th century, Waston first proposed the SR (stimulus-response) theory, which only studied the relationship between external stimulus and response, neglecting the influence of stimulus factors on recipients’ internal cognitive and emotional changes [15]. In line with the deficiencies of SR theory, Tolman proposed that stimuli indirectly influence the body’s response and the body’s internal perception, mediating between stimuli and user behaviors [16]. SOR theory, that is, the “stimulus-body-response” theoretical framework, is a new customer-behavior theoretical model proposed by psychologists Mehrabian and Russell in 1974 [17]. According to this model, external environmental factors (S) can influence individual emotion and cognition (O), thus promoting psychological and behavioral responses (R), as shown in Figure 1. The SOR model has become one of the representative theories for studying consumer behavior, and has been applied to the research into commercial retail and online shopping [18].

The analysis of consumers’ purchase intentions through the S-O-R model shows that the purchase intention is internally and externally affected by factors including consumers’ self-factors (hedonic value, use value, etc.), environmental stimulus, community interaction, product factors, etc. By stimulating consumers’ psychological perceptions, consumers are encouraged to have an emotional state of purchase motivation, and are guided by the motivation to increase their purchase intention, thus leading to purchase behavior.

#### 2.3.2. Perceived-Value Theory

The coming of a new consumption mode has seen an essential evolution of the consumers’ demands and the emergence of a community e-commerce favored by an increasingly growing number of consumers. Perceived value is consumers’ psychological evaluation of the effectiveness of products and services, which underpins the following purchasing behavior. When the products are highly evaluated, the perceived value will be elevated. Zaithaml put forward the theory of perceived value based on the factor of customer perception, which was a consumer’s overall value-assessment of product effectiveness by weighing the cost and benefit. Meanwhile, it was noted that the cost, quality, and credit degree of products or services, as well as the emotional performance of users, were important indicators for measuring perceived value [19]. Sweeney and Soutar built a theoretical model of consumer perceived-value through the four value-dimensions of quality, price, society, and personal emotion, to indicate which consumer values would drive purchase behavior [20]. Scholars’ research on consumer perceived-value highlights the effect of consumers’ cognition of product utility on consumers’ psychological perception and the corresponding responsive behaviors. In community e-commerce, only by satisfying the needs of consumers regarding products and services can they improve the experience and develop the consumer perceived-value, thus making them ready to purchase.

### 2.4. Summary

From the perspective of community e-commerce, in-depth research has been conducted on this new business model and its relationship with traditional e-commerce, from proposing the concept of “community” to expanding community logic to community e-commerce platforms. From the angle of consumers’ purchase intentions, there are representative conclusions from the concept of consumers’ purchase intentions to the in-depth discussion of the internal and external influencing factors on consumers. However, few studies focus on the relationship between these two perspectives. For this reason, this paper will take Xiaohongshu APP, a community e-commerce platform, as an example for searching for the factors that stimulate consumers’ purchase intentions on the community e-commerce platform and to analyze the emotional state of the organism to study the responsive behaviors of consumers’ psychology. Meanwhile, reliable suggestions for the progress of community e-commerce platforms will be given, to prod consumers to purchase.

## 3. Model Hypothesis and Construction

### 3.1. Research Hypothesis

#### 3.1.1. Influence of Product Features on Perceived Value

For the Xiaohongshu platform, price, quality, brand, innovation, and other product features can directly influence consumers’ judgments of product value and their perception of the product value. Steiner argued from the perspective of geographical indication, asserting that it represents the quality of specific products that can create unique consumer perceived-value for geographical-indication products [21]. Based on the customer-purchase evaluation, Wang and Chen explored this and concluded that the popularity of commodity brands within the model of O2O and the price and quality of commodities could also be contributors to consumer perceived-value [22]. Han and Zhang adopted the neuromarketing theory to demonstrate that the quality of product-service brand portfolios would influence the sensitivity of perceived-value of product-service characteristics [23]. Based on the above research and analysis, the following hypothesis is made:

**Hypothesis** **1.** *Product features influence the perceived value positively.*

#### 3.1.2. The Impact of Content Marketing on Perceived Value

The content marketing of Xiaohongshu is a catalyst to encourage consumers to buy goods. Bloggers advertise a product by sharing in pictures or vlogs the product-use experience, travel records, and the process of taking the examinations for the civil service and for postgraduate entrance. When content marketing is closely linked to the products on Xiaohongshu, the quality of the marketing content will constantly influence consumers’ choices. Peters et al., thought that content marketing would encourage consumers to further change their emotional cognition [24]. Zhou remarked from the angle of brand value that content marketing would not only raise consumer participation and perception of brand value but also positively augment consumer loyalty to the brand [25]. He et al., studied content marketing on We Media and pointed out that dialogue, consumer interaction, and storytelling could significantly and positively boost consumer brand-perception and attitude, and that the perceived value of brands presented itself as a mediator between content marketing and brand position [26]. According to Lieb’s research, enterprises should have a good grasp of the marketing content when marketing their products. They need to provide marketing content that can attract consumers and resonate with them [27]. Based on the above research, the hypothesis is proposed as follows:

**Hypothesis** **2.** *Content marketing has a positive influence on perceived value.*

#### 3.1.3. Influence of Community Factors on Perceived Value

The operation mode of the Xiaohongshu platform is characterized by interactive information, emotional connection, a supportive atmosphere, and the key opinions of leaders of the community, which raise consumers’ awareness of the value of products and services and prompts consumers to have a perception of value. Consumers’ participation in the community can satisfy three psychological needs: connection, belonging and identification [28]. Liu et al., pointed out that the interactive information of communities could reduce the purchasing risk, enabling consumers to generate perceived value and making the purchase more possible [29]. Lin et al., remarked that enterprises’ sharing of community-member data could strengthen customers’ awareness of brand information, and the relationship between these two was mediated by perceived value and emotion [30]. Zhi carried out research that focused on fresh agricultural products, and believed that its community marketing could make the consumers more emotionally related to the products, which encouraged consumers to generate perceived value, thus forming a customer group [31]. Based on the above research, this paper makes the following assumption:

**Hypothesis** **3.** *The community factors have improved the perceived value markedly.*

#### 3.1.4. The Influence of Perceived Value on Consumers’ Purchase Intentions

Perceived value refers to the estimated benefit consumers can obtain or the risk they need to bear, which is closely linked to consumer purchase intention. Perceived value is regarded as an important prerequisite factor affecting consumer purchase behavior [32]. On the Xiaohongshu platform, consumers vary in their purchase intentions, due to their different perceived values of products or services. Zhu and Wang studied geographical products, and believed that only by improving the perceived value of users who buy agricultural products with regional symbols online can their purchasing intention be improved, thus expanding into a larger market [33]. Chen et al., studied the traits of famous hosts of the live-streaming platform, and stated that as online celebrities had more noticeable traits, fans’ perceived value could be enhanced, spurring their purchase intention accordingly [34]. Based on the above research and analyses, it can be assumed that

**Hypothesis** **4.** *Perceived value positively influences the consumers’ purchase intentions.*

### 3.2. Construction of Models

**Figure 2:**
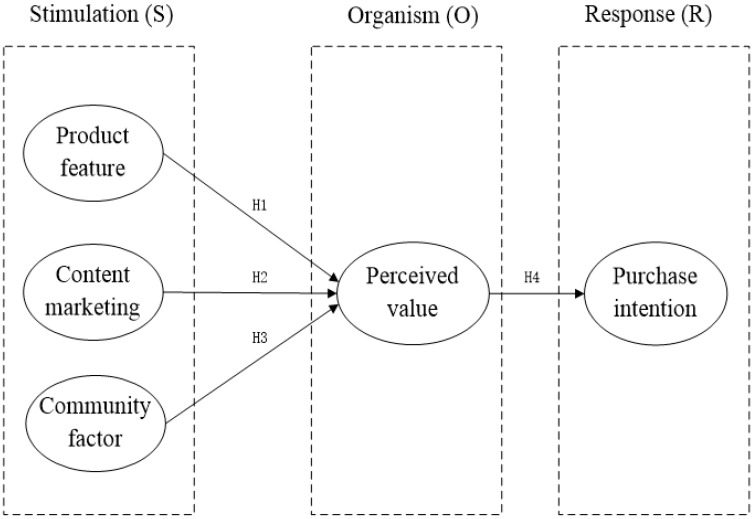
The Model of Factors Affecting Consumers’ Purchase Intentions on Community E-commerce Platform.

The construction of the influencing-factor model of consumers’ purchase intention on the community e-commerce platform is based on the SOR model and combined with the theory of perceived-value, which is obtained by summarizing the existing research results of relevant scholars, in the above research hypothesis. It can be seen that the research hypothesis examines the antecedent variables and consequence variables of perceived value. Therefore, firstly, product features, content marketing and community factors are taken as antecedent variables, and selected as the “stimulus” (S) factor; secondly, consumer perceived-value is selected as the “organism” (O) factor; and again, purchase intention is taken as the consequence variable and selected as the “reaction” (R) factor. This is entirely appropriate. Therefore, this study builds a model of influencing factors of consumers’ purchase intentions on community e-commerce platforms, as shown in Figure 2.

## 4. Questionnaire Design

### 4.1. Measurements

Based on the previous scales, the measurement scales are set according to the actual situation, as shown in Table 1.

### 4.2. Design of Research Scheme

Based on previous scales, this study designs the questionnaire according to its needs and the guidance of the thesis advisor. The questionnaire is divided into a survey for consumers’ personal information and a measurement scale. The first part involves multiple-choice questions that mainly focus on gender, age, educational background, occupation, monthly income, frequency of purchasing products on the Xiaohongshu platform, and the contact time with this platform. The second part introduces the five-point Likert scale, which enables the respondents to choose the answers that apply to themselves more intuitively. It mainly presents the effect of the three independent variables on the Xiaohongshu platform, namely, the product features, content marketing, and community factors, on the perceived value, and the effect of the perceived value on the consumers’ purchase intentions, followed by analyses based on the collected data.

### 4.3. Collection of the Questionnaire Data

This study surveyed the public who used the Xiaohongshu APP from April to May 2022. A total of 366 questionnaires were collected through WeChat moments, QQ Spaces, etc.; 65 invalid questionnaires with short filling times and the same choices were screened out, and 301 valid questionnaires were obtained.

## 5. Statistical Analysis on Influencing Factors of Consumers’ Purchase Intentions on Community E-commerce Platform

### 5.1. Descriptive Analysis

Descriptive analysis of the first part of the questionnaire is carried out through spss 26.0 software to investigate the basic situation of consumers. The results are shown in Table 2.

To sum up, the 301 subjects are evenly distributed by gender, with females slightly outnumbering males. Respondents aged 20–29 with the educational background of undergraduate and junior college, and students, are larger in number compared to other dimensions, which to some extent indicates that young groups with distinct community characteristics are the primary users of the Xiaohongshu APP. From the perspective of income, almost all the respondents who earn CNY 1500 and below or CNY 1501–2500 are students, and most of the respondents in the range of CNY 3501 and above are working populations. As for the platform contact time, the largest number is 2–5 years, accounting for 45.18%, followed by 1–2 years, taking up 30.23%. The number of people who have used Xiaohongshu for less than one year is relatively small, accounting for 13.29%, which also verifies the high popularity of the Xiaohongshu platform from another perspective. However, since the Xiaohongshu APP was launched in August 2014, just more than 7 years ago, fewer respondents have been exposed to it for more than 5 years.

### 5.2. Reliability Analysis

In this study, SPSS 26.0 software is used to test the reliability of all items of the scale and the questionnaire data. The measurement results are shown in Table 3.

It can be seen from the above table that Cronbach’s alpha value of the questionnaire registers 0.942, which represents high reliability. It is manifested that the questionnaire data are reliable and consistent, as the Cronbach’s alpha coefficient-values of each measurement variable are all higher than 0.7, and the total correlation of all corrected items is no less than 0.4.

### 5.3. Validity Analysis

Validity analysis refers to the process of testing the energy efficiency of each item in the questionnaire. This study inspects the validity of the questionnaire data using an exploratory-factor test and aggregate-validity test.

#### 5.3.1. Test of Exploratory Factors

Exploratory-factor analysis is one of the important methods for testing the construct validity of the scale. This paper analyzes the validity of the KMO test and Bartlett’s test of sphericity on the data of three-dimensional items of the independent variables, using spss26.0 software. The measurement results are obtained in Table 4.

The tests show that the KMO value in the test results records 0.917 > 0.6, and the sig value is 0, which is less than 0.001, implying that the research data has passed the validity test. For further validity-tests, factor analysis has been conducted on the items of these three dimensions, the results of which are shown below.

According to Table 5, three factors are extracted through factor analysis, and the items of these independent variables are greater than 0.5, which shows that the extracted three factors are representative. Factor 1 includes four items, i.e., PF1, PF2, PF3, and PF4, corresponding to the product characteristics of the questionnaire; Factor 2 consists of CM1, CM2, and CM3, which correspond to the content marketing; Factor 3 covers CF1, CF2, and CF3, countering the community factors. Furthermore, the rotated squares plus the load of the three factors is more than 70%, which makes the questionnaire easily explicable.

#### 5.3.2. Convergent-Validity Test

This study involves five potential variables, namely, product characteristics, content marketing, community factors, perceived value, and purchase intention. The structural validity of the measurement model is verified through observations and tests of the aggregation-factor load, combination reliability (CR), and AVE values between the variables and potential variables. AMOS 23.0 software is applied in the inspection. The details are illustrated in the following table.

As can be observed from Table 6, there is a factor load of more than 0.5 correspondingly in the perceived value, purchase intention, product features, content marketing, and community factors, which shows that the items that match each potential variable in the questionnaire are of high representativeness. The structural model is proved to have good structural validity and ideal aggregation validity, with the combination reliability of each latent variable all exceeding 0.7, and an AVE value of approximately 0.5.

### 5.4. Correlation Analysis

Correlation analysis is used for the realization of the closeness among realization variables, which is carried out based on the Pearson research in this paper by introducing the SPSS 26.0. The results are shown in Table 7, below:

According to the research hypothesis, this paper mainly explores the correlation between product features, content marketing, community factors and perceived value, as well as the correlation between perceived value and purchase intention. The data results in Table 8 show that all variables have a significant moderate-positive-correlation (ps < 0.01). Therefore, based on the results of the correlation analysis, the structural-equation model can be further used to verify the assumed relationship among variables.

### 5.5. Structural-Model Analysis

#### 5.5.1. Fitting Analysis of the Structural Model

The fitting analysis is used to check the consistency of the theoretical model and the actual data. The structural model is examined by chi-square degrees-of-freedom ratio (X^2^/df), RNSEA, GFI, and other data. The results are as follows:

In the fitting coefficient of the modified model shown in Table 8, X^2^/df is 1.907, which is less than 3; RNSEA is 0.055, under 0.08; GFI is 0.910, exceeding 0.8, and other coefficients are greater than 0.9, indicating that the results of the above fitting-coefficients satisfy the requirements. Hence, the model is ideal for adaptation.

#### 5.5.2. Test of the Structural Model

The following chart shows that the structural model in this study is constructed by Amos23.0, involving five potential and nineteen observation variables.

**Figure 3:**
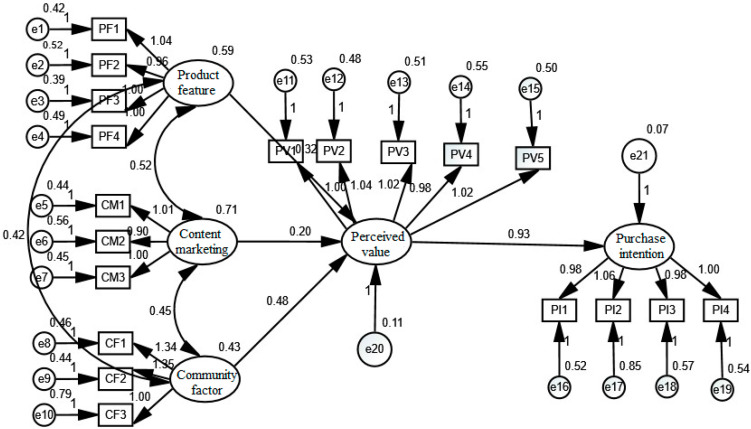
Structural-model chart.

As can be concluded from Figure 3, product characteristics, content marketing, and community factors boost markedly consumer perceived-value and the perceived value, in turn, spurs consumers’ purchase intentions, which is consistent with the above research hypotheses. Through the test of modeling the path coefficient, the degree of positive influence is obtained, as shown in the following table:

According to the analysis results of the path coefficient in Table 9, the path coefficient of product features is 0.317 and *p* is 0.004, which is less than 0.01; that of content marketing is 0.198 and *p* is 0.028, less than 0.05; that of community factor is 0.484 and *p* is less than 0.001. Hence, it can be concluded that the perceived value is significantly advanced by product characteristics, content marketing, and community factors, thus proving the H1, H2, and H3 of the study are valid. The path coefficient of perceived value is 0.932 and *p* is less than 0.001, indicating that perceived value boosts the purchase intention markedly, so Hypothesis 4 is valid. According to the size of the path coefficient, it can be obtained as community factors > product characteristics > content marketing, which complies with the results of correlation analyses.

## 6. Conclusions and Management Inspiration

### 6.1. Research Conclusions

By studying the Xiaohongshu App, this paper takes product features, content marketing, and community factors as Stimulation (S), perceived value as Organism (O), and purchase intention as Response (R), to build a model of consumers’ purchase intentions on the community e-commerce platform. Based on the theory of perceived-value and the SPSS and AMOS, this paper conducts empirical research and analysis to understand the influencing factors of consumer purchase-intention. The main findings are:

#### 6.1.1. Product Features, Content Marketing, and Community Factors Have a Positive Impact on Consumers’ Perceived Value

Product features, content marketing, and community factors have significant positive effects on consumers. In terms of product features, consumers tend to focus on the quality, price, and evaluation of products when they purchase on the platform. Innovative products can attract more consumers. Regarding content marketing, entertainment and practical information can deepen consumers’ understanding of a product, so that they can pay more attention to it. Rich and interesting social-interaction can prompt consumers to participate actively. For the community factors, recommendations from community opinion-leaders and experience-sharing in the process of social interaction all contribute to consumers’ choices. A better mind-evaluation of consumers and a higher level of perceived value can be achieved by offering a more innovative and quality-guaranteed product design and adjustment of the community e-commerce platform by emphasizing the professional and high-quality content-marketing output and the community relations present on the platforms.

#### 6.1.2. Consumer Perceived-Value has a Positive Influence on Consumers’ Purchase Intentions

As an important contributor to consumers’ purchase intentions, perceived value can significantly influence consumers to purchase. A higher psychological perceived-value of platform products means greater purchase intention, and vice versa. In the latter condition, consumers will then turn to other platforms to purchase. When the products on the community e-commerce platform can make consumers feel that the products are worth every penny they spend, consumers’ expectations are satisfied, and the product image is improved, thus impelling the consumers to be willing to buy products.

### 6.2. Implications for Management

Consumer needs have been increasingly diversified with the rapid development of e-commerce. In this context, the community e-commerce platforms that want to surpass others in the fierce market competition have to be good at capturing consumers’ minds. Moreover, they should attach importance to the cost performance of products, enrich content marketing, and closely relate the factors of the community to the products, to improve consumers’ purchasing experiences. At the same time, the continuously evolving technology has urged the community e-commerce platforms to keep up with the development of the times and to explore appropriate marketing models within continuous exploration. Relevant management suggestions are as follows:

#### 6.2.1. Create Utmost Cost–Performance to Make Products Stand Out, thus Enhancing the Perceived Value

The mushrooming of online transactions has led to the emergence of diversified e-commerce platforms, which provides more channels for consumers to compare and purchase products. The utmost cost–performance is indispensable, to mark the products out in the consumers’ choices. Only products with high cost–performance can make consumers feel that the products they purchase are definitely good value for money and then stimulate them to be willing to buy. To win the hearts of consumers, businesses on the community e-commerce platforms need to conduct market research on products in advance to know consumers’ ideas in time, and they have to focus on product adjustment to achieve high cost–performance. While ensuring the leading price and quality of products, it is also urgent to pay attention to the development and innovation of products and after-sales service, and keep abreast of the development trends of the market.

#### 6.2.2. Emphasize Precision-Marketing to Enhance the Authenticity and Effectiveness of the Content and Form of Word-Of-Mouth Propagation

The success of marketing determines whether products and services can be deeply embraced by people. Marketing is the key channel for products to distinguish themselves on the community e-commerce platform. Only by satisfying the consumers’ urgent needs can the marketing content attract them to learn more. To attract consumers’ attention, businesses need to have an insight into consumers’ consumption features, tap into their real needs, focus on innovative marketing-content, enrich entertainment and functional information, and achieve a relative balance between content marketing and users’ needs. In addition, the marketing plan has to be carried out by making full use of consumers’ psychological reactions to establish a long-term stable relationship with customers and then to attract more users, in which way word-of-mouth effect is stimulated for the value realization of content marketing, thus promoting consumers’ purchase intentions.

#### 6.2.3. Enliven the Platform to Improve Community Connection and Initiative

What makes community e-commerce distinct from traditional commerce is community interaction. Businesses are enabled to have close interaction with consumers through community e-commerce platforms. A friendly and authentic community-atmosphere with active interaction can make consumers more willing to share. Consumer feedback will be collected in time to tap into consumers’ diverse needs. Therefore, businesses in the community e-commerce platforms should encourage users to share product and service experiences, and stimulate consumers’ enthusiasm for interaction. The festival promotional activities should be carried out using activities offering interesting experiences. The user experience and evaluation of consumers should be emphasized, so that consumers can gradually form a sense of identity with, and have an enhanced perceived value of, these products. Meanwhile, it is necessary to pay attention to content authenticity and service reliability, through which reputation can be gained to win customers’ trust and stimulate purchase intention, laying the foundation for stable and long-term community management.

#### 6.2.4. Gain User Trust by Supervising the Platform

Recent years have witnessed the rise of community e-commerce that seeks temporary economic benefits from carrying out fraudulent and dishonest practices in the products, services, and marketing content. Hence, the inaction of the platform in areas of supervision will cause a continual loss of consumers. The community e-commerce platform should focus on long-term goals in its development, to strengthen the supervision of the platform, improve the authenticity of the content, lay stress on the examination of content authenticity and reliability, and spread positive and healthy marketing-content. The platforms should effectively safeguard consumers’ lawful rights and interests by cracking down on counterfeits and shoddy goods and launching strict screening-measures for the suppliers, through which they shoulder their social responsibility as enterprises, and consumers’ concerns about product selection can be dispelled.

### 6.3. Research Limitations and Prospects

This research constructs a model of three dimensions to study the influencing factors of consumers’ purchase intentions on Xiaohongshu, one of the community e-commerce platforms. Other platforms, apart from Xiaohongshu, can be included in the subsequent research, by building more perspectives, such as combining the influence of factors of online live broadcasts, which will help achieve a deeper and more thorough understanding of consumers’ purchase intentions on similar platforms.

## Figures and Tables

**Table 1:** Questionnaire.

Measurement Dimension	No.	Items	Reference Source
Product feature	PF1	I think the product quality on the Xiaohongshu platform is guaranteed	Xu and Richmond [35,36]
	PF2	I think the products on the Xiaohongshu platform are cheaper than those offline	
	PF3	I think the products on the Xiaohongshu platform are trustworthy	
	PF4	I think the products on Xiaohongshu are unique and attractive	
Content marketing	CM1	I think consumers are interested in the entertainment information (travel notes, practical and credible content-sharing, live broadcast, etc.) of the Xiaohongshu platform	Zhou [25]
	CM2	I think consumers will like the social interaction on the Xiaohongshu platform	
	CM3	I think consumers are interested in the functional information of the Xiaohongshu platform (product-function introduction, usage method, etc.)	
Community factor	CF1	I have observed that consumers are willing to accept the opinions of community leaders on the Xiaohongshu platform	Xu and Yang et al. [35,37]
	CF2	I am happy to share the experience of using products and services with others on the Xiaohongshu platform	
	CF3	I have used the Xiaohongshu platform for a long time (3 years)	
Perceived value	PV1	The products purchased on the Xiaohongshu platform are worth the money	Wei et al. and Zhang [38,39]
	PV2	The products purchased on the Xiaohongshu platform can meet my expectations	
	PV3	I can get help with the choices of the right product on the Xiaohongshu platform	
	PV4	The products purchased on the Xiaohongshu platform can help me establish a good personal image	
	PV5	The products on the Xiaohongshu platform are more pleasant to the eye	
Purchase intention	PI1	I am ready to open the product page of the Xiaohongshu platform	Xu, Zhang et al. and Pavlou [35,40,41]
	PI2	Among all the community platforms, I prefer to buy goods on the Xiaohongshu platform	
	PI3	The information on the Xiaohongshu platform easily arouses my purchase intention	
	PI4	I often buy things on the Xiaohongshu platform	

**Table 2:** The statistics and analysis table of description.

Item	Options	Frequency	Ratio (%)
Gender	Male	144	47.84%
	Female	157	52.16%
Age	Below 20	55	18.27%
	20–29	125	41.53%
	30–39	77	25.58%
	40-years-old and above	44	14.62%
Educational background	Junior high-school and below	17	5.65%
	High school	40	13.29%
	Junior-college education	88	29.24%
	Undergraduate	127	42.19%
	Postgraduate or above	29	9.63%
Monthly income (monthly living-expenses for students) (Yuan)	1500 and below	49	16.28%
	1501–2500	73	24.25%
	2501–3500	57	18.94%
	3501 or above	122	40.53%
Occupation	Students	125	41.53%
	Government officials	27	8.97%
	Employees	40	13.29%
	Self-employed	38	12.62%
	Other	71	23.59%
Contact time (Year)	Below 1	40	13.29%
	1–2	91	30.23%
	2–5	136	45.18%
	More than 5	34	11.3%

**Table 3:** Reliability Test Results.

Variables	No.	Total Correlation of Corrected Items	Items with Deleted Cronbach’s-Alpha Values	Cronbach’s Alpha Coefficient
Product features	PF1	0.696	0.790	0.842
	PF2	0.667	0.803	
	PF3	0.713	0.783	
	PF4	0.627	0.820	
Content marketing	CM1	0.673	0.706	0.803
	CM2	0.602	0.779	
	CM3	0.674	0.705	
Community factor	CF1	0.667	0.597	0.763
	CF2	0.625	0.646	
	CF3	0.497	0.787	
Perceived value	PV1	0.693	0.815	0.853
	PV2	0.704	0.812	
	PV3	0.673	0.821	
	PV4	0.595	0.840	
	PV5	0.661	0.824	
Purchase intention	PI1	0.596	0.737	0.788
	PI2	0.585	0.747	
	PI3	0.586	0.742	
	PI4	0.628	0.721	
Total measurements				0.942

**Table 4:** Exploratory-factor test results.

Take Samples with Adequate KMO-Measurements		0.917
Bartlett’s test of sphericity	Approximate chi-square	1462.215
	Degree of freedom	45
	Significance	0.000

**Table 5:** Rotated component-matrix.

No.	Component			Rotated Square and Load		
	1	2	3	Total	% of the Variance	Cumulative Percent
PF2	0.845			2.549	25.494	25.494
PF3	0.759					
PF1	0.723					
PF4	0.542					
CM1		0.806		2.457	24.575	50.068
CM3		0.802				
CM2		0.685				
CF3			0.834	2.000	20.001	70.070
CF1			0.681			
CF2			0.600			

Note: Extraction method: principal-component analysis. Rotation method: varimax with Kaiser normalization. Rotation converged in 7 iterations.

**Table 6:** Results of convergent-validity test.

Route	Estimate	AVE	CR
PF1 <--- Product feature	0.775	0.572	0.842
PF2 <--- Product feature	0.715		
PF3 <--- Product feature	0.793		
PF4 <--- Product feature	0.739		
CM1 <--- Content marketing	0.788	0.580	0.805
CM2 <--- Content marketing	0.713		
CM3 <--- Content marketing	0.782		
CF1 <--- Community factor	0.791	0.538	0.775
CF2 <--- Community factor	0.799		
CF3 <--- Community factor	0.592		
PV1 <--- Perceived value	0.722	0.534	0.852
PV2 <--- Perceived value	0.751		
PV3 <--- Perceived value	0.735		
PV4 <--- Perceived value	0.709		
PV5 <--- Purchase intention	0.737		
PI1 <--- Purchase intention	0.716	0.486	0.791
PI2 <--- Purchase intention	0.656		
PI3 <--- Purchase intention	0.699		
PI4 <--- Purchase intention	0.716		

**Table 7:** Analysis result of the correlation.

	Product Feature	Content Marketing	Community Factor	Perceived Value	Purchase Intention
Product feature	1				
Content marketing	0.665 **	1			
Community factor	0.676 **	0.652 **	1		
Perceived value	0.700 **	0.687 **	0.706 **	1	
Purchase intention	0.692 **	0.571 **	0.643 **	0.758 **	1

Note: ** At the level of 0.01 (two-tail), the correlation is significant.

**Table 8:** Fitting coefficient of structural model.

Sample Quantity	X^2^/df	RNSEA	GFI	NFI	CFI	IFI	TLI
Test data	1.907	0.055	0.910	0.913	0.956	0.957	0.949
Adaptation critical-value	<3.00	<0.08	>0.8	>0.90	>0.9	>0.90	>0.90

**Table 9:** Standard-coefficient and hypothesis-test results.

Relations between Variables	Path Coefficient	*p*	Hypothesis Test	Result
Product feature→perceived value	0.317	0.004	H1	Pass
Content marketing→perceived value	0.198	0.028	H2	Pass
Community factor→perceived value	0.484	***	H3	Pass
Perceived value→purchase intention	0.932	***	H4	Pass

Note: *** *p* < 0.001.

## Data Availability

The main data and models generated or used during the study appear in the submitted article; the others are available from the corresponding author, on request.
